# Deep learning-based protoacoustic signal denoising for proton range verification

**DOI:** 10.1088/2057-1976/acd257

**Published:** 2023-05-12

**Authors:** Jing Wang, James J Sohn, Yang Lei, Wei Nie, Jun Zhou, Stephen Avery, Tian Liu, Xiaofeng Yang

**Affiliations:** 1 Department of Radiation Oncology and Winship Cancer Institute, Emory University, Atlanta, GA, United States of America; 2 Department of Radiation Oncology, Northwestern University, Chicago IL, United States of America; 3 Radiation Oncology Division, Inova Schar Cancer Institute, Fairfax, VA, United States of America; 4 Department of Radiation Oncology, University of Pennsylvania, Philadelphia, PA, United States of America; 5 Department of Radiation Oncology, Mount Sinai Medical Center, New York, NY 10029, United States of America

**Keywords:** protoacoustic, signal denoising, Bragg peak, deep learning, stack auto-encoder

## Abstract

Proton therapy is a type of radiation therapy that can provide better dose distribution compared to photon therapy by delivering most of the energy at the end of range, which is called the Bragg peak (BP). The protoacoustic technique was developed to determine the BP locations *in vivo*, but it requires a large dose delivery to the tissue to obtain a high number of signal averaging (NSA) to achieve a sufficient signal-to-noise ratio (SNR), which is not suitable for clinical use. A novel deep learning-based technique has been proposed to denoise acoustic signals and reduce BP range uncertainty with much lower doses. Three accelerometers were placed on the distal surface of a cylindrical polyethylene (PE) phantom to collect protoacoustic signals. In total, 512 raw signals were collected at each device. Device-specific stack autoencoder (SAE) denoising models were trained to denoise the noise-containing input signals, which were generated by averaging only 1, 2, 4, 8, 16, or 24 raw signals (low NSA signals), while the clean signals were obtained by averaging 192 raw signals (high NSA). Both supervised and unsupervised training strategies were employed, and the evaluation of the models was based on mean squared error (MSE), SNR, and BP range uncertainty. Overall, the supervised SAEs outperformed the unsupervised SAEs in BP range verification. For the high accuracy detector, it achieved a BP range uncertainty of 0.20 ± 3.44 mm by averaging over 8 raw signals, while for the other two low accuracy detectors, they achieved the BP uncertainty of 1.44 ± 6.45 mm and −0.23 ± 4.88 mm by averaging 16 raw signals, respectively. This deep learning-based denoising method has shown promising results in enhancing the SNR of protoacoustic measurements and improving the accuracy in BP range verification. It greatly reduces the dose and time for potential clinical applications.

## 1. Introduction

Proton therapy is an ion therapy that has garnered increasing interest in both research studies and clinical applications in radiation oncology. Protons have a unique property where their linear energy transfer (LET) dramatically increases for small velocities, resulting in most of the particle energy being deposited at the end of its trajectory before it is completely stopped in materials. This results in a well-defined dose depth and a highly localized energy deposition in tissues, which is known as the Bragg peak (BP) [1, 2]. The proton therapy provides a more conformal dose and a well-characterized dose depth compared to photon therapy, which exhibits an exponential decrease in dose distribution in deep tissues. Due to the high LET at the end of the range, no or minimal doses are deposited beyond the range [3]. As a result, proton therapy can accurately deliver a high dose to tumors while largely sparing the adjacent organs at risk (OARs).

However, the range verification of the BP within tissues is critical for treatment planning. Several noninvasive techniques, including positron emission tomography (PET) [4, 5] and prompt gamma imaging [6], have been proposed to localize the BP location *in vivo* during real-time radiation therapy [7]. However, both methods require bulky and complex instrumentation and only provide indirect information about the BP position, making it challenging to achieve accuracy in a few millimeters for clinical application.

The protoacoustic determination of BP range has been actively studied for the localization of BP since there is a direct correlation between the acoustic signals and BP locations [8–12]. When a pulsed proton beam deposits energy in a medium, the energy dissipates in fast heat expansion and emits acoustic waves due to thermoacoustic conversion of heat to pressure. It is proposed that the proton range verification can be directly measured based on the time-of-flight (TOF) of the generated acoustic waves. Another advantage of the protoacoustic technique is that it may be used to monitor proton dose distribution in a patient in real-time owing to its relatively simple device-setup compared to PET or prompt gamma imaging. However, it remains a challenging task since the proton acoustic signal is very weak and noisy, and it typically requires delivering a large number of pulses to a single spot in the medium to obtain high-quality proton acoustic signals with a high signal-to-noise ratio (SNR).

Jones *et al* [13] averaged as high as 2048 single signals to obtain stable and accurate measurements, Nie *et al* [14] averaged over 1024 signals, and Kellnberger *et al* [15] used a lower number of 512 averages for their 3D ionoacoustic scans. The large number of signals required to average for the final measurement will result in high doses (e.g., averaging over 2048 proton pulses [13] correspond to 38.9 Gy) to the medium, as well as a long beam delivery time, hindering the clinical application of protoacoustic range verification. If BP can be identified with as few measurements as possible, both the delivery dose and time can be reduced, which is the way forward to the clinic. Therefore, an appropriate denoising method to improve the acoustic signal acquisition is essential to apply the protoacoustic technique in clinics.

In previous studies, denoising techniques such as low-pass filters [16], wavelet-based transformations (WT) [17, 18] and correlation based methods [19] were used to eliminate high-frequency noise in signals. However, these methods rely on prior knowledge such as the choice of thresholding methods or the simulated filter templates, and residual noise that is complex with an unknown frequency domain distribution still needs advanced treatment. Recently, data-driven approaches using deep learning-based methods, such as the stacked autoencoder (SAE) paradigm [20, 21], have shown success in denoising applications, including electrocardiogram (ECG) denoising [22–25]. Our hypothesis is that the proton acoustic signals, which have a similar noise pattern to ECG signals, can also be denoised with an SAE network while minimizing the number of measurements. We used a patch-based method for data augmentation to address overfitting. The long signals were first divided into smaller sections with moving origins and large overlaps, and then fed into the SAEs for denoising. Finally, the denoised small patches were merged by averaging them, resulting in a long-merged signal. We used three detectors to collect proton acoustic signals generated by proton pulses in a plastic phantom and utilized SAEs to denoise the signals while preserving the BP signal with minimized signal acquisitions.

## 2. Methods

### 2.1. Experiment setup and signal acquisition

**Figure 1.**
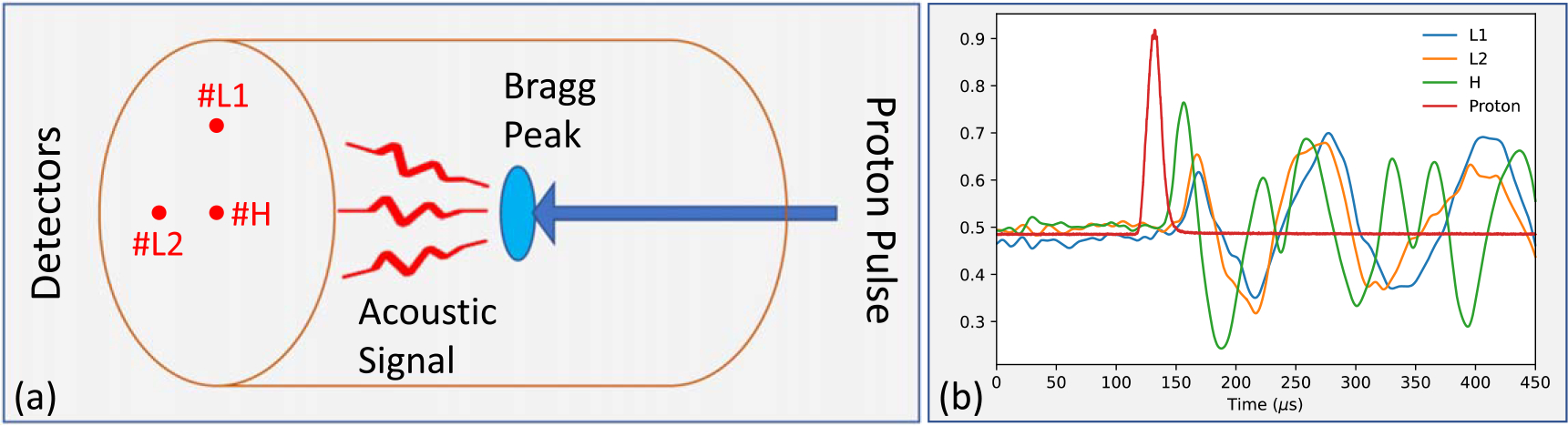
(a) Experimental setup of measuring protoacoustic signals. A 226 MeV proton beam entered the PE phantom and produced a Bragg peak (BP) inside the medium, emitting pressure waves due to thermal expansion. The acoustic signals were collected by three detectors at the other end of PE phantom. (b) The clean signals(NSA = 512) collected at three detectors(L1, L2, and H) and the proton pulses averaged over 512 measurements.

To collect protoacoustic signals, three detectors (accelerometers) were placed on the distal surface of a cylindrical polyethylene (PE) phantom (diameter = 20.88 cm, length = 33.58 cm) as illustrated in figure 1(a). A 226 MeV proton beam was first attenuated by a 2 cm solid water (Gammex 457-CTG, Middleton, WI, USA), and then the proton pulse was incident onto the PE phantom from one end and generated a BP inside the phantom, emitting protoacoustic signals. The acoustic signals were measured by the three detectors placed on the other end. The three detectors are two accelerometers of relatively low accuracy (Type 4374, Denmark) and one accelerometer of high accuracy (Brüel&Kjær, Type-4017-C), namely L1, L2, and H. The electric charge signal was amplified (65 dB, × 1780 gain) and filtered (10 Hz high pass and 100 kHz low pass filter) by a Nexus charge amplifier 2692 before output through a 4-channel digital oscilloscope (Picoscope 5444B, Pico-Tech, UK).

In total 512 proton pulses were incident onto the PE phantom, and each proton pulse would produce a BP inside the phantom. An incident proton pulse has an average current of 490 nA, consisting of 5.7 × 107 protons, which is equivalent to 2.36 cGy of dose delivery to the BP in phantom. For one BP, each of the three detectors would independently collect an acoustic signal emitted from the BP, which is to say, we collected altogether 3 × 512 raw acoustic signals with three detectors and each detector collected 512 raw data. By averaging the 512 single raw signals (NSA = 512), we can obtain a high quality and stable signal for each detector. The averaged signals of three detectors as well as the averaged proton beam are shown in figure 1(b). For easy comparison, all signals have been normalized to [0,1] before averaging.

### 2.2. BP range verification

**Figure 2.**
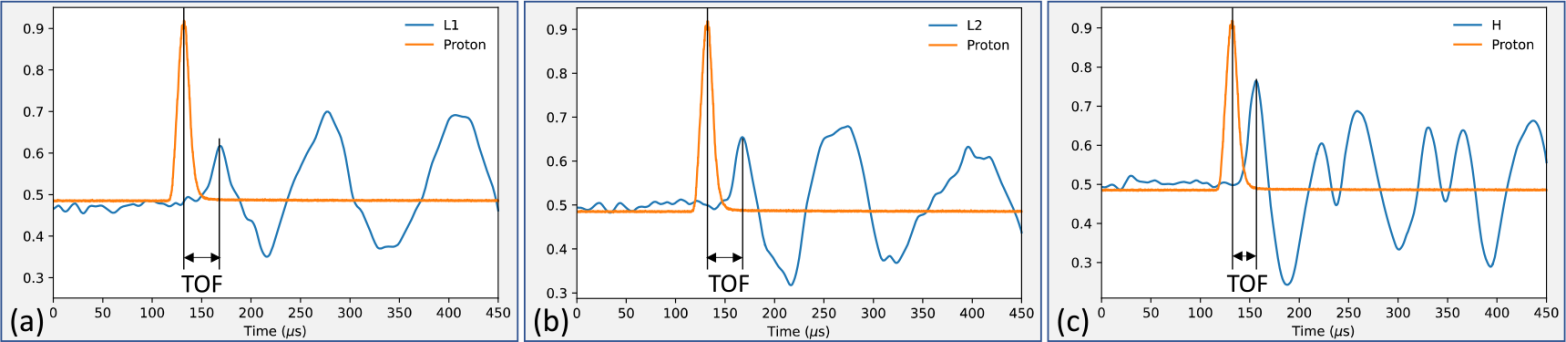
The calculation of time-of-fly (TOF).(a) The proton signals averaged over 512 raw signals(in orange) and the clean protoacoustic signal(NSA = 512) collected at the detector L1. Time-of-flight(TOF) can be measured from the time elapse between proton pulse signal peak and the arrival of acoustic signal (the first peak of the pressure wave). (b) and (c)show the TOFs of L2 and H detectors, respectively.

Protoacoustic is a straightforward technique for measuring the location of the Bragg peak directly by calculating the time-of-flight (TOF) between proton pulses and the arrival of acoustic waves. As shown in figure 2, the TOF is characterized by the time elapsed between the minimum of the proton pulse and the first maximum of the acoustic wave. The BP position can be calculated using the following equation:

(1) $$\tau_{i}=\frac{d_{i}}{c}+\delta_{i},$$

where $\tau_{i}$ is the acoustic TOF for the $i^{\mathrm{th}}$ detector, c is the speed of sound in PE phantom (2.07 mm *μ*s ^−1^), $d_{i}$ is the distance between BP location and the $i^{\mathrm{th}}$ detector, and $\delta_{i}$ is the unique responsive time of each detector.

The value of $\delta_{i}$ can be calibrated through systematic phantom experiments, as described in previous studies [14]. In this work, we are not interested in the calibration of the detector response time. Our goal is to locate the first maximum of the acoustic wave that characterizes the arrival of the acoustic signal. This is because the identification of the acoustic arrival from a noisy signal is one of the most challenging tasks in reducing the number of pulses needed for protoacoustic measurements. However, this task is potentially feasible with the SAE denoising method, as we will demonstrate in the following sections.

### 2.3. Workflow and training strategy

Once we have obtained the 512 raw signals for each detector (L1, L2, and H), SAE networks were trained to reconstruct the denoised signals from a smaller number of raw signals (low NSA). Since each detector is a unique device with specific response times, we trained device-specific models for each detector. For each detector, we randomly selected 256 raw signals for model training and used the remaining 256 raw signals for external testing purposes.

The training dataset for this study involved 256 raw signals, and the grand ground truth (grand GT) was obtained by averaging these signals. To generate the input noisy signals, a small number of raw signals (NSA = 1, 2, 4, 8, 16, and 24) were averaged. Correspondingly, clean signals were generated by averaging 192 out of the 256 raw signals (NSA = 192), which included the raw signals used in the noisy signals. Two training strategies were employed to train denoising models using SAE. The first strategy involved supervised learning, where the input noisy signals were trained with clean signals as the learning target. The second strategy was self-supervised learning (unsupervised), which minimized the reconstruction error between the input noisy signals and the output denoised signals, without reference to the generated clean signals (unsupervised learning) [20].

The differences between the two training strategies would be reflected in the loss function, as discussed in the following section. To evaluate the performance of the SAEs, a similar treatment was applied to the other 256 raw signals in the testing dataset to obtain the grand ground truth (GT) signal, noisy test signals, and clean target signals. After training the SAEs, a new noisy signal from the test dataset was fed into the trained SAE model to obtain the denoised signal. The denoised signal was then compared to the grand GT signal for model performance evaluation.

**Figure 3.**
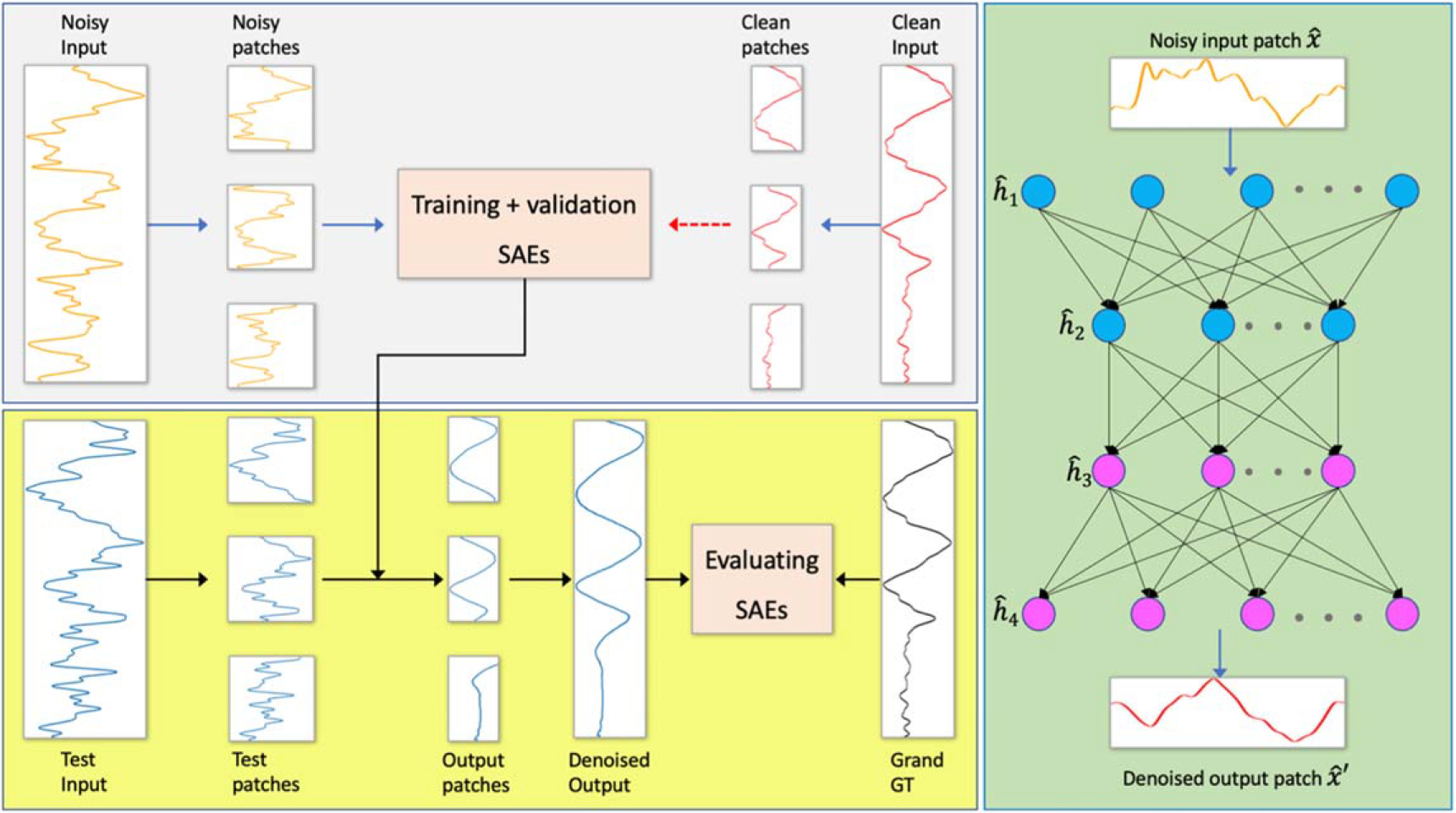
Workflow of denoising protoacoustic signals and the sketched diagram of SAEs.

As a result of limited resources, we performed the protoacoustic measurements at only one energy level of 226 MeV, resulting in highly limited patterns of acoustic waves, essentially containing only three intrinsically independent patterns due to the three detectors used. Considering the whole long signals (more than 450 *μ*s) as training objectives could lead to overfitting. To address this issue, we trained our SAEs in a patch-based manner by cutting the long signals into smaller sections with shifting origins. This allowed us to greatly augment the size and variation of the training and testing datasets. The reconstructed long signals were obtained by merging the SAE output patches. Figure 3 sketches the workflow of denoising the acoustic signals.

### 2.4. Network architectures

The architectures of the proposed SAEs are shown in figure 3, consisting of a compressing encoder path and an expanding decoding branch. All the one-dimensional input noisy signals are first normalized into [0,1] and cut into small patches as $\overset{ˆ}{x}$. Similarly, the patches of corresponding training clean signals $\overset{ˆ}{y}$ were also obtained. Through encoder layers, the input $\overset{ˆ}{x}$ is encoded to output $\overset{ˆ}{h}$ in the hidden layers. This step serves as extracting higher-level useful representations of the signals and eliminating the redundant noisy components of the input. Then, the useful latent features $\overset{ˆ}{h}$ reconstrcut to the output ${\overset{ˆ}{x}}^{\prime }$ via decoder layers. After that, the aim of SAEs is to minimize the reconstruction error either between ${\overset{ˆ}{x}}^{\prime }$ and $\overset{ˆ}{y}$ for supervised training, or between ${\overset{ˆ}{x}}^{\prime }$ and $\overset{ˆ}{x}$ for unsupervised training.

Denoting $\theta_{i}=\left\{w_{i},b_{i}\right\}$ are the weighting and bias parameters for the $i_{\mathrm{th}}$ SAE layer and s(x)=1/(1+exp(-x)) is a sigmoid activation function to introduce nonlinearity between SAE layers, the hidden output can be represented as

(2) $$\begin{array}{l} {\overset{ˆ}{h}}_{1}=s\left(w_{1}\overset{ˆ}{x}+b_{1}\right),\\ {\overset{ˆ}{h}}_{i}=s({w_{i}}^{\prime }{\overset{ˆ}{h}}_{i-1}+b_{i}),\;i=2,\;3,\;4. \end{array}$$

Denoting $\theta^{\prime }=\left\{w_{\mathrm{out}},b_{\mathrm{out}}\right\}$ are weighting and bias parameters for the last decoder layer, we define the decoder’s output as

(3) $${\overset{ˆ}{x}}^{\prime }=s(w_{\mathrm{out}}{\overset{ˆ}{h}}_{4}+b_{\mathrm{out}}).$$

For each denoised signal ${\overset{ˆ}{x}}^{\prime }$, it is reconstructed form the noisy signal $\overset{ˆ}{x}$, and is expected to resemble either the clean signals $\overset{ˆ}{y}$ (supervised) or the input $\overset{ˆ}{x}$ (unsupervised). Thus, for supervised training the learnable parameters are optimized by minimizing the reconstruction error:

(4) $$\theta_{i},\theta^{\prime }=arg\;\underset{\theta_{i},\theta^{\prime }}{min}\;{\overset{ˆ}{x}}^{\prime }-{\overset{ˆ}{y}}_{2}^{2},\;\mathrm{or}$$

for unsupervised training, the learnable parameters are optimized by minimizing the reconstruction error:

(5) $$\theta_{i},\theta^{\prime }=arg\;\underset{\theta_{i},\theta^{\prime }}{min}{\overset{ˆ}{x}}^{\prime }-{\overset{ˆ}{x}}_{2}^{2}.$$

where the L2 norm aims to minimize the difference between ${\overset{ˆ}{x}}^{\prime }$ and the learning target $\overset{ˆ}{x}$.

The unsupervised method proposed in this study uses the exact same SAE architectures as the supervised method. The only difference is that in unsupervised learning, the loss function measures the similarity between the noisy input and the denoised output, rather than between the ground truth and denoised output. This means that the unsupervised method doesn’t require ground truth to supervise the learning process, hence its name.

### 2.5. Experiment design and training parameters

To perform the experiments mentioned above, we conducted multiple experiments for both supervised and unsupervised training, with different NSAs for input noisy signals. During the training phase, 100 long noisy input signals were generated for each NSA of 1, 2, 4, 8, 16, or 24. We used a patch size of 66 *μ*s with an origin shift of 3.2 *μ*s for data augmentation. After data augmentation, we obtained 13,400 signal patches for training. Among the 13,400 patches, two-thirds of the data was used for training, and one-third was used for validation of the SAE models.

### 2.6. Model evaluation and BP range verification

Two metrics were used to measure performance: mean squared error (MSE) and signal-to-noise ratio (SNR):

(6) $$MSE=\frac{1}{n}\sum_{i=1}^{n}\;{\left(s_{i}-x_{i}\right)}^{2},$$

(7) $$SNR=10\;{log}_{10}\frac{\sum_{i=1}^{n}\;x_{i}^{2}}{\sum_{i=1}^{n}\;{\left(s_{i}-x_{i}\right)}^{2}},$$

Where $s_{i}$ and $x_{i}$ are the long grand GT signal and long noisy/denoised signals from the test dataset (the remaining 256 raw signals outside the training dataset), respectively. Both MSE and SNR were computed for noisy and denoised signals, to evaluate the efficacy of SAEs.

Additionally, we evaluated the BP displacement between the pre-training and after-training signals by calculating the TOF differences based on the grand ground truth (GT). As discussed in previous sections, this work focuses on identifying the first maximum of the pressure wave, which characterizes the arrival of the acoustic signal. We used the first peak of the grand GT (averaged over all 256 raw signals) as the reference time point of the acoustic arrival. For simplicity, we automatically located the maximum between 0 and 192 μs of a merged long acoustic signal to identify the arrival of the first pressure peak. For both noisy and denoised signals, their first peaks would have shifts from the reference time point. The shift of time of flight (TOF) multiplied by the sound speed (approximately 2.07 mm *μ*s^−1^ in the PE phantom used in the experiments) corresponds to the uncertainty of BP range verification.

To estimate the BP range uncertainty before and after denoising, we used two metrics, mean error of the shifts (ME^BP^) and mean absolute error of the shifts (MAE^BP^). This step is helpful to quantitatively examine the feasibility of applying the SAE denoising in clinics.

### 2.7. Model performance compared to other denoising methods

The wavelet transformation (WT) and low pass filters (i.e., Gaussian low-pass filters) are widely used denoising techniques [17, 26]. They have been extensively studied and developed, and there are now readily available software packages for implementing these methods in Python and other programming languages. Due to their popularity and ease of use, these tools are often the first choice for researchers and practitioners working with noisy signals and data. To demonstrate the superior performance of our proposed SAE models, we will perform denoising on the test datasets using both WT and Gaussian low-pass filters, and compute the MSE, SNR, and BP location errors to quantitatively compare the performance of our method against other methods.

The wavelet transform (WT) operates in the frequency domain and is effective at removing high-frequency noise from signals, similar to the well-known fast Fourier transform (FFT). However, unlike the FFT, the WT has the advantage of providing multi-resolution analysis and improved time resolution, making it suitable for non-stationary signals. To perform the WT, we used the ‘PyWavelets (pywt)’ package [27] in Python. We employed the Daubechies 4 (db4) wavelets for multilevel (level = 11) one-dimensional decomposition and reconstruction of the long protoacoustic signals. To obtain the best denoising results with the highest SNRs, we iteratively eliminated various levels (level = 1 to 11) of the decomposed coefficients until we achieved optimal denoising performance.

On the other hand, the Gaussian low-pass filter is a popular classical signal denoising tool by convolving the signal with a Gaussian kernel, which is a discrete approximation of the Gaussian function. The filtered output is then a smoother and less noisy signal. We used the ‘scipy.ndimage’ package in the SciPy library [28] to perform Gaussian low-pass filtering. The Gaussian kernels with multiple standard deviations (sigma = 1.6, 3.2, 6.4, 12.9, 19.3, 25.8, 38.7, or 51.6 *μ*s, remembering the patch size we used for SAEs is 66 *μ*s) and a truncation of 4.0 were used, and the best results with highest SNRs were recorded. We optimized the classical models in a supervised manner by maximizing the SNRs (equivalent to minimizing the MSEs) of reconstructed signals relative to the ground truth, so the recorded best denoising performance of the WT and low-pass filters could be directly compared with the supervised SAEs. All the algorithms were implemented using Python version 3.9.12 [29] in this paper.

## 3. Results

### 3.1. Evaluation of SAEs on the test dataset

**Figure 4.**
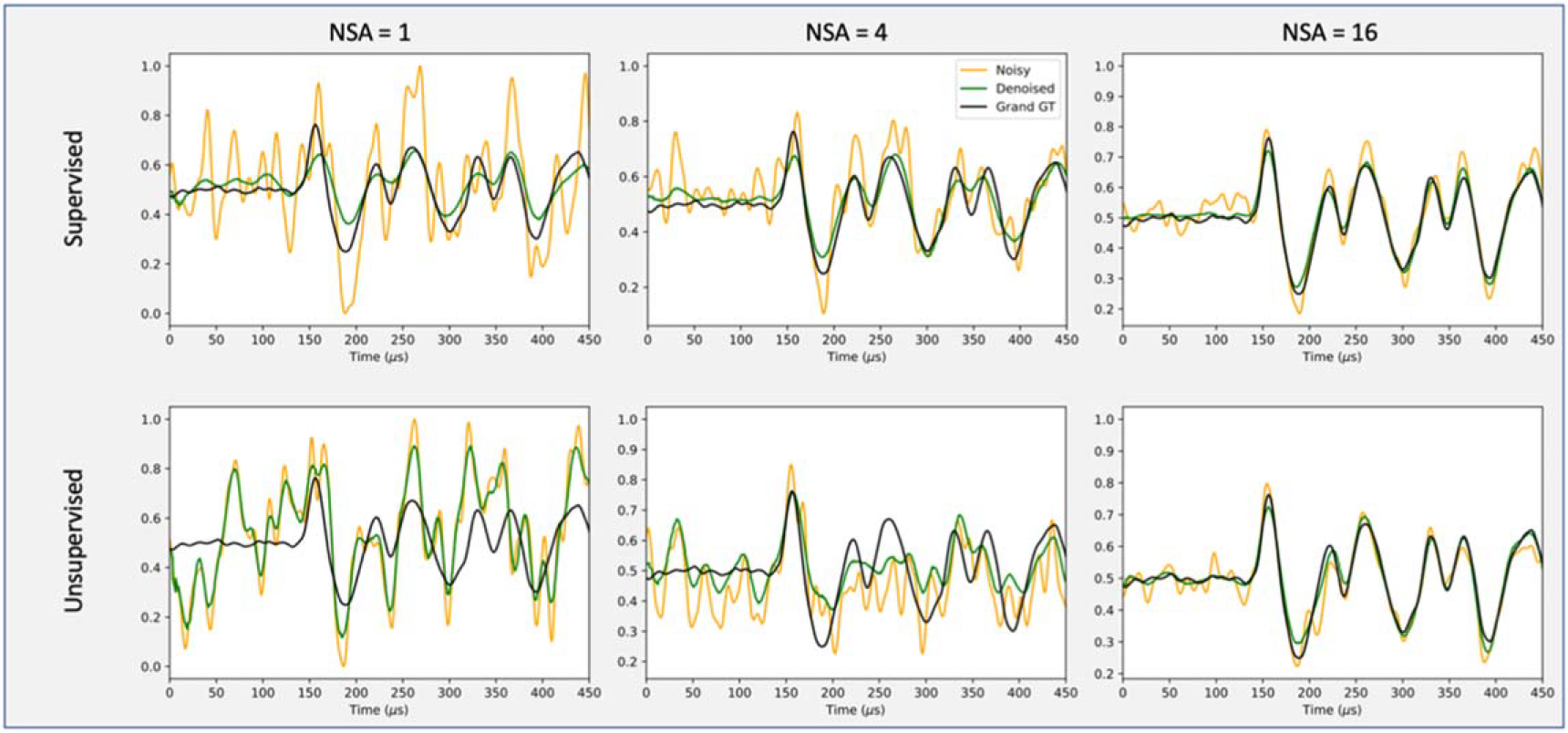
Examples of the noisy input signals(orange curves), reconstructed denoised signals(green curves), and grand GT signals (black curves) obtained on the high accuracy detector(H).

Table 1 shows the MSE and SNR values of L1 detector signals before and after denoising on the test dataset, using the supervised training strategy. The metrics are calculated from the first 400 *μ*s of merged long signals. Table 2 shows the MSE and SNR changes in percentage after denoising, for various experiments on the test set. Figure 4 shows the example noisy input signals, reconstructed denoised signals, and the grand GT signal of H detector, for both supervised and unsupervised learning. For both training strategies, the MSE values of after-denoising were dramatically reduced while the SNR were enhanced. Moreover, at low NSA = 1, 2, and 4, supervised learning outperformed unsupervised learning in both metrics.

### 3.2. BP range uncertainty

From our experiments, the BP uncertainties began to show significant improvement at an input NSA greater than 2 for detector H, and at an input NSA greater than 4 for detectors L1 and L2, considering the largely decreased standard deviation of ME^BP^ and MAE^BP^. Table 3 shows the ME^BP^ and MAE^BP^ representing BP range verification uncertainties before and after denoising, with NSA starting at 4. The values were converted to millimeters by multiplying the TOF peak shifts with the sound speed in the PE phantom (2.07 mm *μ*s^−1^).

**Figure 5.**
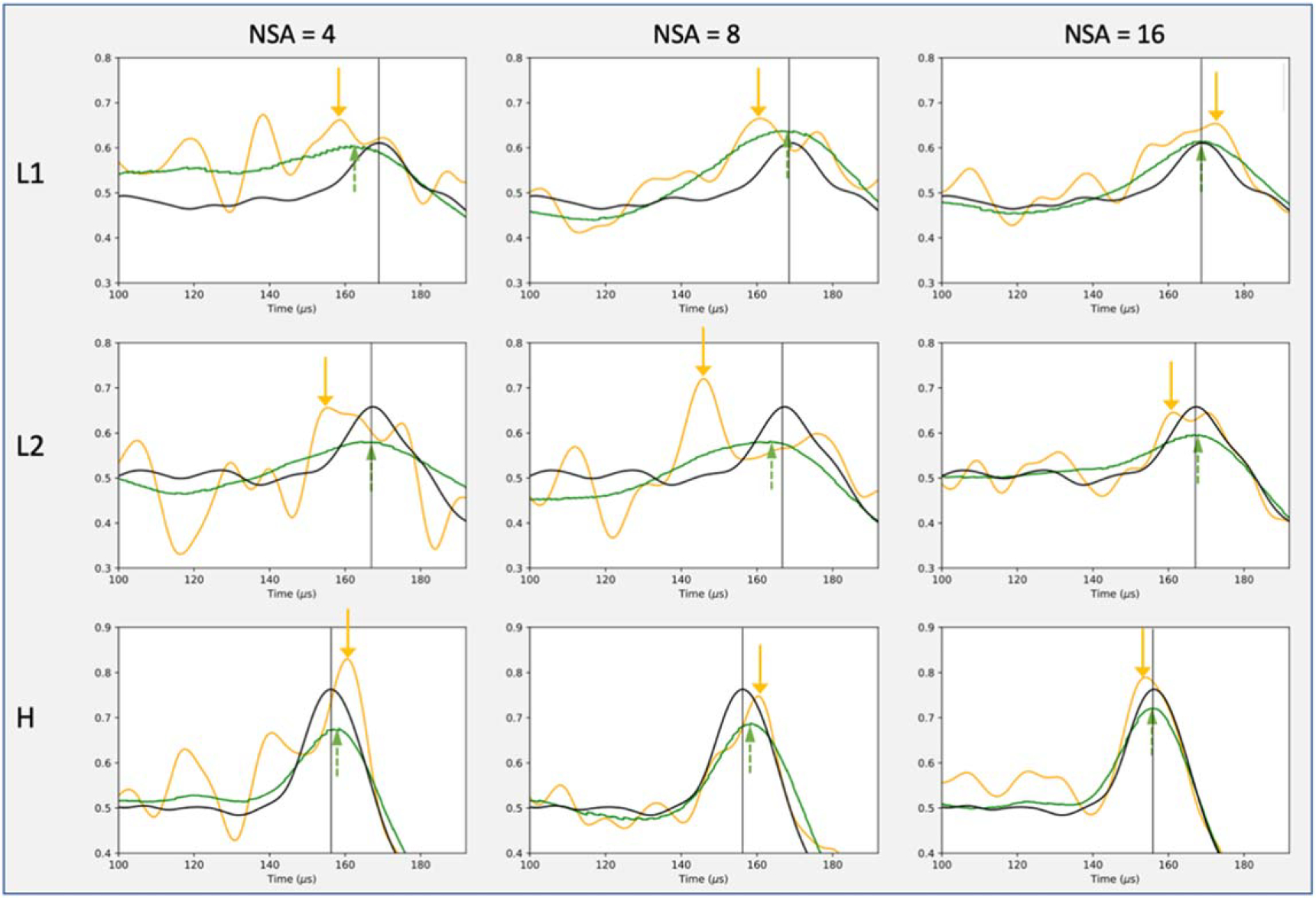
Examples of improved BP verification after supervised learning denoising. The three columns represent noisy input signals of NSA = 4, 8, and 16. The signals were collected on the three detectors L1, L2, and H, and zoomed in to the range between 100 and 192 *μ*s. The orange arrows indicate the BP position on the noisy signals(orange curves), while the green arrows indicate the denoised BP position. The black vertical lines denote the baseline BP position from the grand GT (black curves). The green arrows are closer to the black baseline compared to the orange arrows, indicating smaller BP range uncertainties.

Figure 5 shows examples of enhanced BP locations after supervised denoising with NSAs of 4, 8, and 16, on all three detectors. To better observe the correction of peak localization, we zoomed in on the range between 100 and 192 *μ*s in figure 5. Among the three detectors, the high-accuracy detector H was the most precise in identifying BP positions, with the least range uncertainties. Furthermore, as the NSA of the raw signals in the input increased, the BP range verification improved both before and after denoising.

### 3.3. Compare with wavelet transform and Gaussian low-pass filter

The performance of the proposed supervised and unsupervised SAEs in denoising were compared with other commonly used denoising methods, such as the WT and Gaussian low-pass filter. Table 4 presents the percentage improvement in SNR and MAE of the BP location for all four methods. The results showed that the supervised SAEs outperformed both the WT and Gaussian filter in terms of SNR enhancement across all experiments. Moreover, the supervised SAEs were found to be more accurate and robust in BP location identification, especially for data with lower NSA (i.e., NSA = 4 or 8). However, the BP localization error for very low NSA (1 and 2) data was not shown in table 4, as none of the three denoising methods improved the BP localization error for such data with high levels of noise. Hence, these results were omitted from the table. For the unsupervised SAE, it outperformed the WT and Gaussian filter in terms of SNR enhancement for NSA >= 8, though it doesn’t always show more accurate BP verification than the WT and low-pass filter.

**Figure 6.**
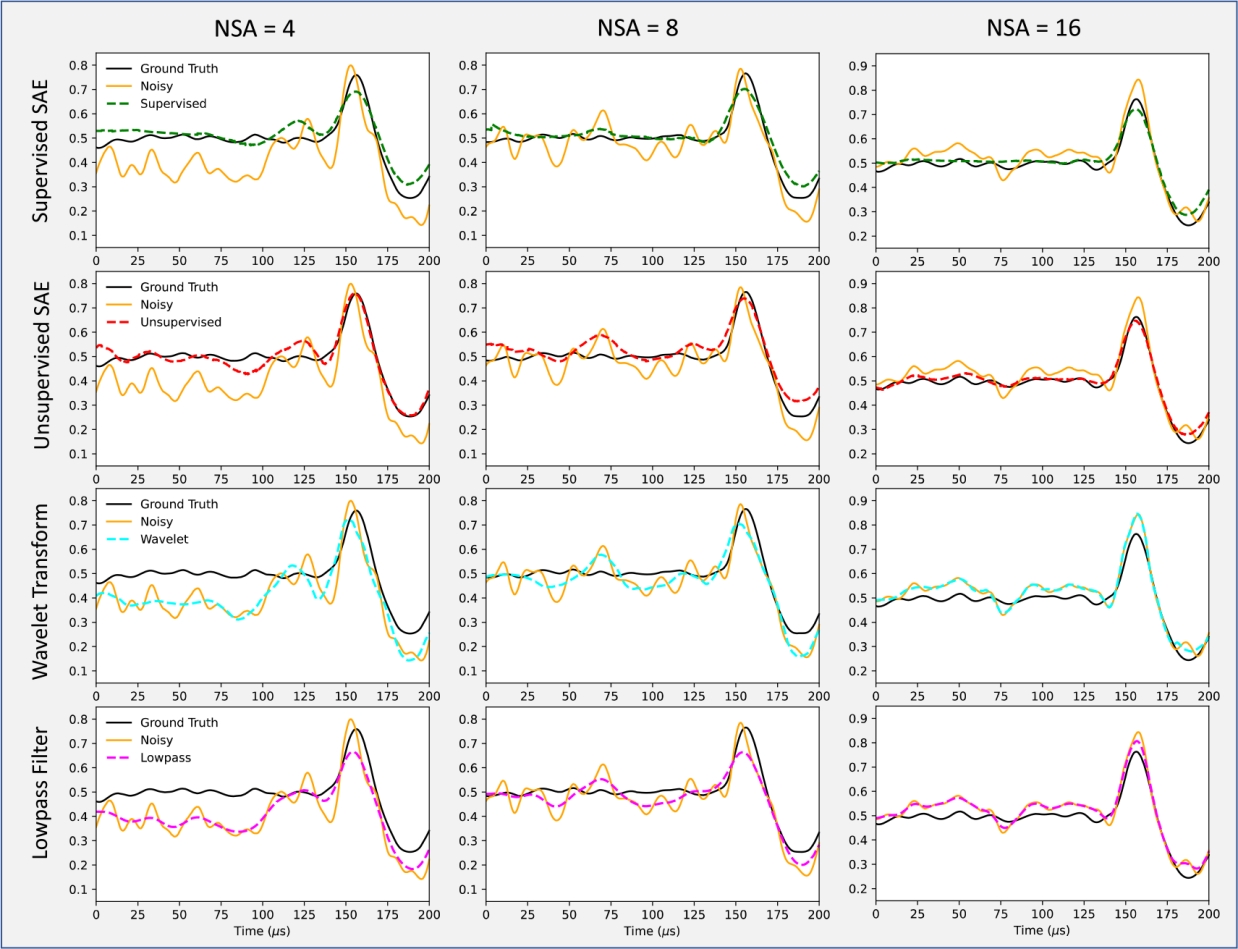
Examples of improved BP verification after supervised SAE, unsupervised SAE, wavelet transformation (WT), and low-pass filter denoising for comparison. The three columns represent noisy input signals of NSA = 4, 8, and 16. The signals were collected on the high accuracy detector(H). The first main peak on the spectrum characterizes the BP range.

Figure 6 depicts examples of improved BP verification after supervised SAE, unsupervised SAE, WT, and low pass filter for comparison. The data were collected using the high accuracy detector (H) and then zoomed in to capture the first main peak on the protoacoustic spectrum between 0 and 200 *μ*s, which could characterize the BP range. It shows that the SAE denoised spectrum are close to the ground truth, while the WT and low-pass filter denoised results are closer to the input noisy spectrum. Such difference is prominent in both the flat section (0 to 125 *μ*s) and the main peak (125 to 175 *μ*s).

## 4. Discussion

The feasibility of the protoacoustic method for *in vivo* BP range verification has been actively investigated. Despite its unique advantages, such as a relatively simple instrumentation setup and straightforward correlation between the pressure/acoustic wave and BP localization, the key measuring objectives, acoustic signals, are susceptible to various sources of noise (thermal, scattering, and internal electric [12, 30, 31]). As a result, a large number of measurements (high NSA) are required to achieve high SNRs, which leads to high dose delivery in tissue and makes it unsuitable for clinical use. To address this issue, we have proposed a deep learning denoising method based on SAE network, which can reconstruct clean signals from only a small number of measurements (low NSA). Thus, our proposed method optimizes BP range verification by reducing the localization uncertainty.

We tested two training strategies with SAE networks, namely the supervised and unsupervised SAEs. Both models achieved a substantial reduction of MSE and enhancement of SNR for low NSA and provided high-quality denoised signals. From table 2, for supervised denoising, the MSE was reduced by about 80%–85% at NSA of 1 and about 47%–68% at NSA of 24 on three detectors, and the SNR increased by 59%–87% at NSA of 1 and 10%–21% at NSA of 24, showing a steadily weaker enhancement of the signals for noisy inputs with larger NSA. This trend correlates with the fact that using more raw signals for average yields more stable and accurate signals. However, this trend is almost reversed for unsupervised models. According to table 2, the MSE reduction and SNR increase were worse for low NSAs of 1, 2, and 4, but better for high NSAs of 8, 16, and 24. Such differences between supervised and unsupervised models arise from the training philosophy behind them.

SAE is a successful denoising tool and is often used for unsupervised learning without any reference labels [20, 32–34]. Unsupervised SAEs try to reconstruct the input signals rather than any given ground truth. As shown in figure 4, when NSA equals to 1, the input signal was very noisy and lacking any obvious mode. Unsupervised SAEs had no knowledge of underlying patterns and would simply treat those noises as some embedded truth to mimic during denoising, producing an output carrying those noises. However, supervised training is a learning strategy that knows the clean ground truth. It was able to distinguish those noisy components in low NSA signals. As NSA increased from 1 to 16, the input signals began to contain more information and show obvious underlying patterns. Thus, unsupervised SAEs were also able to capture representative features apart from the random noises, and both unsupervised and supervised SAEs achieved similar results. In many SAE denoising applications, the ground truth labels are missing or unavailable. But, in this specific denoising task, we were lucky to have the high NSA signals serving as ground truth. Therefore, we were able to change the training strategy from unsupervised learning to fully supervised learning and achieved better results, especially for signals with small NSAs.

The results of comparing the proposed supervised SAEs with classical denoising tools such as WT and Gaussian low-pass filters are very promising. The supervised SAEs outperformed both methods in terms of SNR enhancement for all experiments, as shown in table 4. The unsupervised SAEs also outperformed both classical methods with higher SNR enhancement for NSA >= 8 cases. This demonstrates the superiority of SAEs in denoising protoacoustic signals due to their strong ability to extract high-level latent features to accurately distinguish and remove noisy components. The large number of hidden neurons in an SAE allows it to learn complicated features with great flexibility, whereas WT and Gaussian filters only deal with a handful of predetermined or handcrafted features. This is a significant advantage of SAEs over classical methods for denoising protoacoustic signals.

In this study, to make a fair comparison with the proposed supervised SAEs, we have optimized the parameters in WT and Gaussian low-pass filter models in a supervised manner to maximize the SNRs between the denoised results and the ground truth, as defined in equation (7). By using the same loss function, we can directly compare the results of supervised SAE, WT, and Gaussian lowpass filter as shown in table 4, where the supervised SAEs generally provide more precise BP localization from denoised signals compared to the two classical methods, especially for relatively low NSA (4 or 8) cases. For the unsupervised SAE method, it used a different loss function and obtained no knowledge from the ground truth, so it yielded worse BP localization in some cases, compared to the other three supervised methods.

One of the most important tasks in protoacoustic measurement is to localize BP in tissues. From table 3, we see that the BP range uncertainty represented by MAE^BP^ and ME^BP^ was substantially reduced after SAE denoising, especially the standard deviations of MAE^BP^ and ME^BP^. Comparing the three detectors, overall, the high accuracy detector (H) performed better than the two low accuracy detectors (L1 and L2), both before and after denoising. Moreover, in most cases, the supervised SAEs yielded smaller or comparable BP location errors than the unsupervised SAEs, demonstrating an overall better performance. After supervised denoising, for the high accuracy detector, the ME^BP^ improved from −10.93 ± 40.36 mm to 0.68 ± 5.54 mm for NSA = 4 raw signals, and improved from −2.12 ± 22.52 to 0.20 ± 3.44 mm for NSA = 8 raw signals, while for detectors L1 and L2, the BP uncertainty at NSA = 8 are well above 5 mm. For L1, the ME^BP^ improved from −12.09 ± 37.88 mm to 1.44 ± 6.45 mm by averaging 16 raw signals. For L2, it improved from −6.48 ± 25.65 to −0.23 ± 4.88 mm for NSA = 16. Such dramatic improvement suggests that with the aid of deep learning techniques, it is possible to achieve accurate BP range verification with just a few acoustic signals, which brings down the dose and time required in protoacoustic measurements.

Previous literature has reported BP range uncertainty in both simulation and experimental studies, ranging from submillimeter to 5 mm [15, 30, 35–37], with some extreme cases reporting a standard deviation of up to 10 mm [36]. Moreover, Jones *et al* found that the BP range uncertainty depends on the proton pulse width [12]. For an extremely narrow Dirac-delta-function-like (FWHM < 4 *μ*s) proton pulse, the systematic error of BP determination is <2.6 mm. However, for longer non-*δ*-function-like beams (e.g., FWHM = 56 *μ*s), a systematic error up to 23 mm can be expected. The desired proton pulse width for typical medical proton cyclotron is around ~10–14 *μ*s, and in our experiment, the proton beam used was modulated by a function generator, achieving ~14–18 *μ*s pulse width (FWHM). The expected BP range uncertainty should be around a few millimeters, much greater than the optimal submillimeter records [30], which is consistent with our denoised results. When treating a patient with proton beams, typically a 3.5% of the range plus 1–3 mm margin is allowed to account for the uncertainty of BP range [36], corresponding to an uncertainty of 3–5 mm in our experiment, considering the BP depth is around 50–60 mm. Our denoised results of the high accuracy detector fall well within that range, and the low accuracy detectors fall marginally in that range.

The main idea of this work is to reduce dose delivery while maintaining the accuracy of BP range verification. In our measurement, each raw acoustic signal was generated by a proton pulse equivalent to 2.36 cGy, so the dose delivery would be 1208.3 Gy to average 512 signals. After denoising, we can obtain BP localization with 16–24 averages for the two low sensitivity detectors and with only 4–8 averages for the high accuracy detector, corresponding to 37.8–56.6 cGy and 9.4–18.9 cGy, respectively. The required doses for BP range verification were significantly reduced with the aid of deep learning denoising techniques.

This paper reports a pilot study that investigates the feasibility of applying deep learning techniques to denoise protoacoustic signals for Bragg peak range verification. This is a novel approach since previous works mainly used frequency domain filters, such as wavelet transformation. Our proposed method uses a stack auto-encoder (SAE) for denoising and demonstrates promising results.

One major limitation of this study is that the proposed methods have been implemented on Bragg peaks produced with only one specified energy of 226 MeV, and incident on a uniform PE phantom, while in clinics, patient tissues are heterogeneous and behave more complicated than the phantom, and the beam energy used for treatment may vary. For now, we are only able to work with a relatively simple dataset due to limited experiment resources, and the experiment conditions are same for the training and testing data. To overcome the potential overfitting problem due to this limitation, we have utilized a patch-based method for data augmentation. Since the patch size (66 *μ*s) is much smaller than the signal size (450 *μ*s), these patches only capture a small section of the spectrum and greatly enrich the data variation. Besides, we have optimized all the denoising models with a supervised manner, to ensure a fair comparison between the classical denoising tools and our proposed supervised SAEs. In this pilot study, the concept of utilizing deep learning to solve the long-standing challenge of BP range verification has been well demonstrated. In future work, we can test the methods on more beam energies and more realistic settings. This will help to further validate the effectiveness and generalizability of our proposed approach.

Besides, as we discussed in the above paragraphs, the proton beam we used was not *δ*-function-like pulses and could intrinsically increase the BP range uncertainty. Additionally, the high accuracy detector outperformed the low accuracy ones in all metrics. To push for better results in the future, possible solutions include using narrower proton pulses as the dose deposition source and sticking to the acoustic wave detectors of higher accuracy.

## 5. Conclusions

In this study, we demonstrated that SAE networks can be used to denoise protoacoustic signals for BP range verification. In addition to the commonly used unsupervised training strategy, we introduced full supervised learning using the averaged signals (NSA = 192) as the training ground truth and achieved better results. We observed decreased MSEs and increased SNRs for all experiments after SAE denoising. For BP range verification, the high accuracy detector outperformed the low accuracy ones and the supervised SAEs outperformed the unsupervised models. Deep learning denoising techniques can be integrated into data acquisition for signal processing during protoacoustic measurements, reducing the dose and acquisition time required for obtaining stable and clean signals.

## Figures and Tables

**Table 1.** MSE and SNR values of L1 detector signals before and after denoising on the test dataset, using the supervised training strategy.

NSA	MSE			SNR (dB)		
	Before	After	Change (%)	Before	After	Change (%)

1	3.39E-02	5.29E-03	−84.38	9.08	16.99	87.21
2	1.62E-02	3.01E-03	−81.41	12.06	18.98	57.40
4	8.00E-03	1.92E-03	−76.03	15.03	21.02	39.87
8	3.81E-03	9.62E-04	−74.74	18.19	24.19	33.03
16	1.85E-03	4.93E-04	−73.28	21.24	26.99	27.04
24	1.22E-03	3.88E-04	−68.24	27.96	27.96	21.22

**Table 2.** MSE and SNR changes in percentage (%) after denoising for all experiments on the test set.

Detector	NSA	MSE (%)			SNR (%)		
		Supervised	Unsupervised	*p*-value^a^	Supervised	Unsupervised	*p*-value

L1	1	−84.38	−28.36	< 0.001	87.21	14.81	< 0.001
	2	−81.41	−36.09	< 0.001	57.40	13.46	< 0.001
	4	−76.03	−52.69	< 0.001	39.87	20.51	< 0.001
	8	−74.74	−52.57	< 0.001	33.03	17.75	< 0.001
	16	−73.28	−57.61	< 0.001	27.04	18.10	< 0.001
	24	−68.24	−68.03	0.63	21.22	21.80	0.64
L2	1	−85.07	−32.46	< 0.001	85.44	14.37	< 0.001
	2	−78.49	−38.92	< 0.001	53.24	16.20	< 0.001
	4	−74.35	−50.61	< 0.001	39.40	21.19	< 0.001
	8	−44.37	−63.73	< 0.001	16.33	25.10	< 0.001
	16	−58.56	−63.80	0.04	18.75	21.18	0.14
	24	−50.58	−57.27	< 0.001	13.09	16.47	< 0.001
H	1	−79.46	−28.13	< 0.001	59.03	12.99	< 0.001
	2	−71.09	−33.34	< 0.001	41.85	15.04	< 0.001
	4	−64.60	−53.61	0.001	26.40	18.86	< 0.001
	8	−65.14	−60.09	0.07	23.29	20.67	0.08
	16	−67.16	−66.10	0.42	21.82	21.72	0.76
	24	−46.59	−60.15	< 0.001	10.48	16.43	< 0.001

a The *p*-values are computed using a T-test to compare the denoising performance of supervised and unsupervised Stacked Autoencoders (SAEs), based on the Mean Squared Error (MSE) or Signal-to-Noise Ratio (SNR) of the denoised data.

**Table 3.** The mean absolute error (MAE^BP^) and mean error (ME^BP^) evaluating BP range uncertainties before and after denoising, for NSA>=4.

Training Strategy	Detector	NSA	MAE^BP^ ± Std (mm)		ME^BP^ ± Std (mm)	
			Before	After	Before	After

Supervised	L1	4	55.90 ± 70.87	41.00 ± 62.20	−48.82 ± 75.92	−32.52 ± 67.03
		8	32.08 ± 57.96	6.32 ± 8.41	−25.40 ± 61.19	−0.45 ± 8.41
		16	16.04 ± 36.38	5.41 ± 3.80	−12.09 ± 37.88	1.44 ± 6.45
		24	5.13 ± 4.27	4.48 ± 3.21	−0.32 ± 6.67	1.06 ± 5.41
	L2	4	51.15 ± 70.02	28.95 ± 57.53	−43.87 ± 74.80	−18.48 ± 61.70
		8	27.45 ± 52.72	12.95 ± 35.35	−22.27 ± 55.10	−5.48 ± 37.25
		16	9.28 ± 24.77	3.95 ± 2.88	−6.48 ± 25.65	−0.23 ± 4.88
		24	5.75 ± 13.30	3.32 ± 2.55	−0.66 ± 14.48	0.50 ± 4.16
	H	4	16.22 ± 38.54	4.60 ± 3.16	−10.93 ± 40.36	0.68 ± 5.54
		8	6.74 ± 21.60	2.84 ± 1.95	−2.12 ± 22.52	0.20 ± 3.44
		16	3.15 ± 2.31	2.10 ± 1.78	−0.31 ± 3.90	0.46 ± 2.71
		24	2.30 ± 1.89	1.86 ± 1.59	−0.11 ± 2.98	−0.64 ± 2.36
Un-supervised	L1	4	39.97 ± 59.81	33.75 ± 54.82	−30.64 ± 65.00	−23.73 ± 59.84
		8	33.77 ± 58.52	31.59 ± 60.45	−28.20 ± 61.39	−26.30 ± 62.93
		16	12.24 ± 28.00	9.63 ± 23.96	−5.84 ± 30.00	−1.91 ± 25.75
		24	6.43 ± 14.27	4.26 ± 3.32	−3.12 ± 15.34	−0.14 ± 5.40
	L2	4	43.05 ± 64.22	40.86 ± 67.31	−33.41 ± 68.73	−33.67 ± 71.18
		8	30.33 ± 56.63	15.22 ± 41.37	−23.29 ± 59.88	−8.74 ± 43.20
		16	6.38 ± 13.34	4.02 ± 3.14	−0.86 ± 14.77	1.17 ± 4.98
		24	3.67 ± 3.30	3.52 ± 2.55	−0.88 ± 4.86	1.06 ± 4.22
	H	4	13.60 ± 35.77	6.50 ± 14.03	−8.37 ± 37.34	−1.91 ± 15.34
		8	6.43 ± 21.69	3.12 ± 2.40	−1.71 ± 22.56	0.07 ± 3.93
		16	3.24 ± 2.35	1.99 ± 1.73	−0.40 ± 3.99	0.32 ± 2.62
		24	2.43 ± 1.99	1.93 ± 1.14	0.10 ± 3.14	0.11 ± 2.24

**Table 4.** Comparison of the denoising performance of the supervised SAE, unsupervised SAE, wavelet transformation (WT) and Gaussian low-pass filter on the same test data. Evaluations are based on the percentage improvement in signal-to-noise ratio (SNR) and the mean absolute error in BP range uncertainties (MAE^BP^).

Detector	NSA	SNR (%)				MAE^BP^ ± Std (mm) After denoising			
		Supervised SAE	Unsupervised SAE	Wavelet transform	Gauss-Lowpass	Supervised SAE	Unsupervised SAE	Wavelet transform	Gauss-Lowpass

L1	1	87.21	15.06	46.73	50.45	—	—	—	—
	2	57.40	14.68	35.51	35.75	—	—	—	—
	4	39.87	14.49	23.51	24.89	41.00 ± 62.20	57.74 ± 72.80	77.18 ± 48.42	50.12 ± 66.40
	8	33.03	19.67	16.84	18.97	6.32 ± 8.41	24.43 ± 50.36	12.57 ± 40.08	25.73 ± 51.30
	16	27.04	21.62	13.04	12.49	5.41 ± 3.80	6.38 ± 11.14	5.66 ± 18.00	10.96 ± 24.21
	24	21.22	19.33	8.38	9.47	4.48 ± 3.21	6.65 ± 16.24	4.23 ± 18.46	5.62 ± 15.98
L2	1	85.44	13.23	42.95	50.83	—		—	—
	2	53.24	16.55	26.46	33.38	—		—	—
	4	39.40	19.87	20.55	24.51	28.95 ± 57.53	38.84 ± 62.20	28.09 ± 61.17	44.43 ± 69.77
	8	16.33	21.84	14.99	16.80	12.95 ± 35.35	14.71 ± 33.83	17.59 ± 50.85	33.18 ± 63.71
	16	18.75	18.26	8.46	11.44	3.95 ± 2.88	4.12 ± 3.23	1.24 ± 1.68	5.27 ± 15.57
	24	13.09	14.37	6.26	9.23	3.32 ± 2.55	3.17 ± 2.41	1.00 ± 1.79	3.56 ± 3.24
H	1	59.03	13.22	17.58	32.11	—		—	—
	2	41.85	11.43	11.74	20.68	—		—	—
	4	26.40	17.37	8.09	13.75	4.60 ± 3.16	7.42 ± 19.09	17.32 ± 32.74	10.21 ± 30.96
	8	23.29	11.18	5.12	9.72	2.84 ± 1.95	5.33 ± 21.68	8.09 ± 2.96	2.79 ± 2.07
	16	21.82	21.50	1.35	4.89	2.10 ± 1.78	3.12 ± 1.64	2.45 ± 1.74	2.29 ± 1.79
	24	10.48	13.37	1.10	4.06	1.86 ± 1.59	1.81 ± 1.53	1.86 ± 1.12	1.78 ± 1.42

Note: For NSA = 1 and 2, none of the four methods improved the MAE^BP^ after denoising, so the values are not shown here.

## Data Availability

The data cannot be made publicly available upon publication because they are not available in a format that is sufficiently accessible or reusable by other researchers. The data that support the findings of this study are available upon reasonable request from the authors.
